# The long-term transformation of the concept of CSR: towards a more comprehensive emphasis on sustainability

**DOI:** 10.1186/s40991-021-00063-9

**Published:** 2021-08-11

**Authors:** Hildegunn Mellesmo Aslaksen, Clare Hildebrandt, Hans Chr. Garmann Johnsen

**Affiliations:** Department of Working Life and Innovation, University of Agder, Jon Lilletunsvei 9, 4879 Grimstad, Norway

**Keywords:** CSR, Sustainability, Social model, Discourse, Business-society relationship

## Abstract

This article adds to the discussion of the long-term transformation of CSR, presenting a perspective on the interplay between CSR debate and public discourse on business responsibility. 50 years after Milton Friedman’s provoking claim that the only responsibility for business is to seek profit, a broader debate has emerged aligning CSR with an increasingly comprehensive concept of sustainability. We trace this evolution of the concept during the last three decades focusing on the intersection of economic, social, and environmental responsibility. Based on discourse analysis of news articles and opinion pieces in the largest public newspaper in Norway from 1990 until 2018, the study confirms that discussions on CSR, sustainability and the social model often approach the same challenges. We argue that sustainability has become the dominating term in popular usage for describing the relationship between business and society. Based on our analysis of the public debate, CSR has become a *more comprehensive term,* transformed from being a term mainly related to internal business affairs to part of a broader societal discussion about sustainability.

## Introduction

Corporate Social responsibility (CSR) has been in the focus for several decades, making it a natural object of reflection and review. Lately, a growing literature has emphasised the long-term transformation of the concept (Carroll, 2021; Idowu et al., 2017; Latapí Agudelo et al., 2019; Matten & Moon, 2020; Windsor, 2021). In a recent review of the historical change in the CSR discourse, Carroll (2021) identifies environmental concern as one of the main themes already in the 1960s. Referring back to Rachel Carson’s, 1962 book *Silent Spring,* both society and business has been addressing common challenges facing society. However, more than half a century on, the debate about the impending ecological crisis and how we should organise our societies is still raging. There is increasing consensus that a further transformation of the business/society relationship is necessary, however, there is little agreement on what such a transformation should look like (Farla et al., 2012; Haberl et al., 2011; Westley et al., 2011). While accepted as a vital part of the complex modern society, business is depicted both as the reason for the crisis and the solution to it (van den Broek, 2020). In this climate of diverging opinions some argue that CSR should shift its focus to a stronger emphasis on sustainability (e.g., Carroll, 2021; Rank & Contreras, 2021; Trollman & Colwill, 2021; Windsor, 2021) and that today’s corporations are conceived as vehicles for change (Matten & Moon, 2020).

Despite the recent focus on the long-term transformation of CSR in academic debate, few have studied the transformation in the public debate. We agree with the claim put forward by Latapí Agudelo et al. (2019) that the evolution of the CSR concept cannot be linked to academic contributions only. As social expectations of corporate behaviour have changed, so has the concept of CSR. Therefore, logically, CSR discourse should be seen in relation to socio-political context and other prominent and related discourses (Latapí Agudelo et al., 2019; Mark-Ungericht & Weiskopf, 2007; Matten & Moon, 2008, 2020; Wehrmeyer et al., 2019). Using Norway as a case, we therefore analyse discourse within CSR, the social model and sustainability, the aim being to capture important intersection between economic, social and environmental responsibility expectations and discussions.

The goals of this study is to 1) examine the transformation of the CSR discourse in the public sphere and 2) interpret how and to what extent the concept of CSR has been influenced by the changing understandings of sustainability and the Norwegian social model during the last three decades. Since our empirical study focuses on public, not academic discourse, the term CSR refers to a wider set of discussions on social responsibility of business. Our main finding is that in the long-term transformation of CSR, sustainability and environmental concerns have become more central to CSR, and that CSR discourse is increasingly merged into the sustainability discourse. This has led to the somewhat paradoxical situation, the discussion on CSR in the public has faded during the last decade, but expectations of business to be responsible have not.

## Positioning our argument in the CSR debate

A substantial number of contributions have critically proposed that the way CSR is framed contributes to enhancing problems rather than solving them. Frequently cited, Banerjee (2008) claims that the CSR discourse is defined by narrow business interests and an emancipatory rhetoric. In the same vein, a noticeable number of authors find that CSR discourse is pervaded by focus on profit, performance and economic values (e.g., Allen & Craig, 2016; Baden, 2016; Brei & Böhm, 2014; Brooks, 2010), shaped by increasingly narrow managerialist perspectives (Marens, 2010), global corporate power (Gilberthorpe & Banks, 2012; Sklair & Miller, 2010) and embedded in a global neo-liberal discourse (Mark-Ungericht & Weiskopf, 2007). Other critical contributions have tended to focus on the academic discourse or business communication and reporting (e.g., Brei & Böhm, 2014; Ellerup Nielsen & Thomsen, 2007; Fuller, 2018; Nwagbara & Belal, 2019; O’Connor & Gronewold, 2013), bringing CSR discourse analysis closer to ‘greenwash’ literature (Gatti et al., 2019) or toward a more general critique of the current economic system. In a more positive strand of CSR scholarship, discourse is given a central role in providing CSR a moral legitimacy (e.g., Christensen et al., 2013; Reynolds & Yuthas, 2008; Seele & Lock, 2015).

In our study, we use media texts to analyse CSR transformation. Resent research suggests that media is an important country-level determinant of CSR activities and a component of national social context (El Ghoul et al., 2019; Hartmann & Uhlenbruck, 2015). Media is considered an important third party that forms and reflects public opinion about business responsibility (Burke, 2021). Nevertheless, research on CSR discourse in traditional news media is rare and varied in method and context (Buhr & Grafström, 2007; Carroll, 2011; Dickson & Eckman, 2008; Herzig & Moon, 2013; Lee & Riffe, 2019; Lunenberg et al., 2016; Madsen & Stenheim, 2014; Tang, 2012).

Summing up, literature on CSR discourse and transformation has tended to assess the discourse from the business perspective. By exploring CSR discourse transformation from the public perspective, our research advances the literature and contributes to current conversations on how CSR is constructed in a socio-political context. Given its social orientation, CSR is influenced by wider movements in the social realm. In the context of Norway, where implicit (Matten & Moon, 2008) and intrinsic (Wehrmeyer et al., 2019) CSR is anticipated, effort is wisely applied by trying to understand the nature and nurture of such implicit-ness and intrinsic-ness. We argue that seeing CSR from the social rather than the business perspective makes even more sense in such a context. By including adjacent and sometimes overlapping discussions on the social model and sustainability, we believe that important public sentiments and expectations towards business are attended to. To our knowledge, no one has performed a qualitative longitudinal study of the transformation of CSR which also takes the formative effects of the far-reaching discourses on the social model and sustainability into account. The originality of the paper thus lies in its wide scope and public perspective.

Our analysis suggests an increasingly comprehensive public CSR thinking. In order to demonstrate this, we divide the discussions into three dimensions, depth, width and level. *The depth discussion* is illustrated by Archie Carroll’s (1991) pyramid model, where the minimum requirement is for business to follow legislation. In later reflections, Carroll (2016, 2021) argues for extending the discussion of the pyramid. *The width discussion* is illustrated by John Elkington (1994) and the concept of Trippel Bottom-line (TBL). Elkington argued that corporate and society interests could be mediated when business incorporated society standards in their own governance perspectives. Thus, social, environmental, and economic concerns should be balanced at business level. Recent research on TBK and integrated reporting has problematised the possibility of balancing these concerns, and rather call for stronger emphasis on certain concerns (Idowu & Del Baldo, 2019). *The level of analysis* discussion can be illustrated by the fact that CSR discussions are referring to United Nations (UN) commission on the common future in the 1980’s, the Paris agreement in 2015 and to the current European Union (EU) initiative, Green Deal. However, when referring to a higher level, our intention is not to shift the debate from business to society or to the political system, it is to refer the business discussion and its integration with social and political discussions. It implies that businesses acknowledge their impact and responsibility, both as individual businesses and as business communities, on the development of society and legislation, what we in the continuation will refer to as the social model. Carroll (2021) refers to this as political CSR.

These three aspects of CSR can be traced back to the discussion of Milton Friedman’s seminal 1970 article in The New York Times Magazine *A Friedman Doctrine: The Social Responsibility of Business is to Increase Its Profits* (Friedman, 1970). In a recent discussion marking 50 years since the article (Sorkin, 2020; Strine & Zwillinger, 2020), several commentators addressed the different dimensions of Friedman’s argument. Friedman’s main point was that business should concentrate on what they do best, which is to produce products and services in an effective way to a market adjusted price. Under fair conditions, profit will be a good proxy for their contribution to social wellbeing. The fairness of the marked conditions is then left to the political system as the regulatory power. Even if logical, this argument neglects several issues.

Firstly, the border between business and political authority is not as sharp as Friedman assumes. Businesses lobby in policy issues and are consulted when new regulations are discussed. Furthermore, business exploit common goods, like air, water, land, and people. History can be used as an indicator that political regulations have not been able to curb these exploitations. Perhaps even more important is the fact that in order to solve the sustainability challenge, a dialogue between business, society and the political system is needed (Strine & Zwillinger, 2020). Still, we acknowledge that different socio-political contexts play into these discussions, exemplified with the term liberal marked economy versus coordinated market economy (Banerjee, 2008; Freeman & Dmytriyev, 2017; Looser, 2019). The table below tries to identify some positions on the discussion.

The table shows the gradual development from a restrictive view of CSR towards a more comprehensive view. Table 1 takes the Friedman doctrine (the upper left) as the minimum position of CSR, as we move downwards and towards the right, more width, and more levels as well as a deeper understanding of CSR are added. The left-hand column is a discussion within what Banerjee (2008) describe as the economic paradigm. Here, CSR is discussed from the perspective of business. It is therefore marked by what has been termed extrinsic CSR (Wehrmeyer et al., 2019). In the right-hand column, the context is more in direction of a coordinated market economy (Looser, 2019) At the same time, we are likely to find more intrinsic CSR in this context (Wehrmeyer et al., 2019). Acknowledging the proposal that there are different national interpretations of CSR (Idowu & Filho, 2009) we utilise the features of our case to contribute to the general debate on CSR in context. Furthermore, in the right column the CSR discourse is shifted from business perspective to business environment and society. Our study of the Norwegian discourse confirms a transformation towards the comprehensive understanding of CSR in the bottom right corner, indicating that society’s expectations towards the business community are far from Friedman’s, making CSR an ambitious guiding principle. Furthermore, we argue that there is a development over time, which shows how the concept of CSR has been transformed and increasingly aligned with the larger societal debate on sustainability.

**Table 1:** Degree of comprehensiveness of CSR thinking

	Business complies with the social and political regime	Business engage in improving the social and political regime
Economic responsibility	Friedman Doctrine: Business must comply with the social and political regime, but otherwise operate with profit. Deeper argument: business share profit with society.	Markets should be competitive. Deeper argument: There should be a dialogue between society and business. Marked-based incentives should stimulate/induce sustainable behaviour of business.
Economic responsibility + social sustainability	Business provides decent work and can develop new ways of work. Deeper argument: Business is the forerunner of social reform, anti-racism, and the like.	Society sets standards for work and support business through the welfare model. Deeper argument: Business should be engaged in taking responsibility for those outside work-life.
Economic responsibility + social and environmental sustainability	Business can help developing green growth technology and solutions. Deeper argument: Business sustain from non-environmentally friendly behaviour.	Business is proactive in promoting environmental restrictions and social wellbeing. Deeper argument: Business becomes activists for sustainability transformation.

## CSR, sustainability, and the Norwegian socio-political context

As an exponent of a coordinated market economy, Norway is a case of a country that embraces a comprehensive discussion of CSR, including a recognition of the interrelatedness of business and society**.** Since CSR discourse is analysed in relation to discourses on the social model and sustainability, the following section briefly presents the theoretical foundation for these concepts. Our analytical focus on transformation and on depth, width, and level purports that we see the concepts as dynamic terms that can reflect different meanings and values, and that these change over time.

### Analysing CSR in Norway

That the concept of CSR is dynamic is well described in Latapí Agudelo et al. (2019). CSR must not be understood as a singular, static concept, but as an ensemble of practices that differ across nations and business systems (Maon et al., 2017). These practices are dependent on historical institutional set-ups, and socio-political drivers contribute to shaping the CSR concept. In the Nordic countries, CSR has been introduced to the context of advanced welfare states emphasising universal rights and duties, extensive state engagement in negotiations and agreements on labour relationships. This model contrasts with fundamental principles of the neoliberal Anglo-American emphasis on corporate discretion, voluntarism, and market-based solutions (Midttun, 2018; Strine & Zwillinger, 2020) and represents an illustrative example of a context for intrinsic CSR. Matten and Moon (2008) introduced the distinction between *explicit* and *implicit* CSR, where the implicit form has been the most common in European countries, also in Norway. The *implicit* form describes corporations´ roles within the wider formal and informal institutions for society’s interests and concerns. It consists of values, norms and rules that often result in codified and mandatory obligations and is motivated by the societal consensus on what is legitimate to expect from *all* major groups in society. Simplified, the implicit CSR is not so much the articulated voluntary decision of a company, but a tacit reflection of norms and expectations in their social surroundings, implying a more comprehensive view of CSR. The *explicit* form describes voluntary corporate strategies and programs that are motivated by the perceived expectations of different stakeholders of the corporation.

It can be proposed that CSR, as a managerial concept has been somewhat considered ‘old news’ in Norway.^1^ The corporate structure, dominated by small and medium enterprises (SMEs) has implied little distance between managers and employees and between companies and society, encouraging a stakeholder orientation without necessarily labelling it CSR or any other equivalent term (Ihlen & Hoivik, 2015). Strand et al. (2015) point to such deep-seated traditions of stakeholder engagement when claiming that the concept of ‘creating shared value’ originates in Scandinavia. Thus, we assume that a more implicit CSR implies a more intrinsic CSR. This means that it is likely that CSR is imbedded as tacit understanding in the context (Looser, 2019), thereby posing a challenge when we are to analyse the public discourse on CSR.

However, even if there is this dominance of a coordinated context in the Nordic countries, we do not believe that the divide between a liberal marked economy and a coordinated marked economy is a sharp one. There is also continuous development of these contexts. In example, Maon et al. (2017) find that even though the Nordic (Denmark, Netherlands,^2^ Norway and Sweden) way of doing CSR is still characterised by consensus and participation, where CSR issues and social concerns are grafted onto the roots of business activities and involving a broad range of stakeholders, CSR is becoming increasingly explicit. In contrast, Tench et al. (2018) suggest that explicit CSR within Europe has become more implicit. Other research has suggested that explicit or implicit CSR has more to do with company size (Kumar et al., 2021). This shows that dual typologies come with limitations.^3^ Thus, the newly proposed extension to include *explicitisation* and *implicitisation* of CSR (Matten & Moon, 2020) may serve better to explain the dynamic and complex properties of the business/society relationship. However, a transformation into more explicit CSR is not necessarily accompanied by change in practice (Mette Morsing & Spence, 2019). Consequently, we take an open-ended approach to CSR discourse in the public sphere. As such, we do not aim to explain CSR better, but to reveal how it has been understood and presented.

### The Norwegian socio-political context

Considering the relevance of the institutional context when studying the CSR perspective (Wehrmeyer et al., 2019), it is necessary to look more closely at the public discourse within the specific institutional context of Nordic capitalism. The “Norwegian social model” is a term that points to formal institutions and agreements alongside more informal and intangible traits. As a term and the concept is dynamic and as resonant as it is inaccurate (Witoszek & Midttun, 2018). Originally in the development of the Norwegian social model, there were three main components influencing the debate; the first being a political desire for a democratic work-life, the second being a social culture for egalitarian structures and mutual participation and thirdly the desire for a competitive advantage which could be accomplished through employee participation (Johnsen & Ennals, 2012). In line with the main features of social models of the other Nordic countries, the Norwegian social model is founded on a tripartite collaboration between the State, labour unions and an employer representative council, where employee representation and national economic and political intervention create an institutionalised intertwining between the business world and social welfare. It has been proposed that founding CSR principles of shared value and industrial democracy originate in the institutional culture of the Nordic countries (Olkkonen & Quarshie, 2019). Contemporary CSR has been introduced to the context of advanced welfare states emphasising universal rights and duties, extensive state engagement and negotiations and agreements in labour relationships. There is a clear contrast to the neoliberal Anglo-American emphasis on corporate discretion, voluntarism and market-based solutions (Midttun, 2018). The model also involves a tendency to focus on competitive advantage through collaboration (Strand & Freeman, 2015). When Norwegian work-life is analysed in relation to the conditions for change, observations depict a cultural inclination towards egalitarian organisational structures and institutionalised employee participation, creating a foundation that is contributary to industrial relations (Gjølberg, 2010; Morsing et al., 2007). Witoszek and Midttun (2018) set out to explain this cultural inclination and its role in the evolution of the model, emphasising the dynamic between cooperation and competition. This has created a cooperative ethos making sure that capitalist profit-seeking agents are always counterbalanced by strong ideals stressing public-mindedness and social cooperation. Thus, the model places several CSR related issues outside the boundaries of the firm. This homogenic picture of ethical harmony is however seen to be somewhat nuanced when the Nordic countries participate on an international level, Kinderman (2020) proposes that the competitive advantage of CSR is the overriding criteria for SME’s and the heterogeneity of Nordic countries materialized in their cautious support of supranational EU regulation.

That which makes the case especially interesting is the complex and often paradoxical interplay between welfare, a well-performing business sector and care for the environment. The Norwegian wealth and welfare system has been mainly funded by the income from the oil and gas industry, which means that welfare in Norway is dependent on fossil fuels. A large number of Norwegian companies are engaged in the fossil industry since the 1970s, in fact, 98% of Norwegian municipalities are home to an oil and gas worker (Statistics Norway 2017), and the industry represents 42% of total export revenue (Statistics Norway 2018). The oil and gas industry has made Norway one of the top five richest countries in the world by Gross domestic product (GDP) (nominal) per capita (The International Monetary Fund (IMF) and World Bank). The tax revenues from the fossil industry have contributed to the building up of the world’s largest sovereign wealth fund with a market value over 1000 billion United States (US) dollars in 2020, in popular called the Oil Fund.^4^

Obviously, oil and gas does not represent a single explanation to the Norwegian wealth and welfare, as all Nordic countries^5^ rank among the world’s wealthiest nations with high levels of welfare without notable fossil industries. Common characteristics of the countries´ national histories, cultures and values has made the entire region a highly competitive periphery, making the Nordic version a specific and successful form of capitalism (Jes Iversen & Thue, 2008) and a nucleus of strong CSR and sustainability performances (Strand et al., 2015). From these descriptions, a more comprehensive CSR thinking can be expected. However, some of the same institutional traits that seem to make a good foundation for combining economic wealth and social welfare with CSR and sustainability, are presented to prevent radical environmental transformation (Dryzek et al., 2002; Midttun & Olsson, 2018; Midttun & Witoszek, 2018). In pursuing sustainable development in ‘the land of Brundtland’, Lafferty et al. (2007) concluded that the Norwegian profile on sustainable development was “long on promise” and “short on delivery” and attributed this to the political competition over economic and welfare benefits fostered by the exceptional growth in public revenues from the oil and gas industry.

### Sustainability

The term sustainability is a contested term that has shifted in meaning before and during the period we study. Kuhlman and Farrington (2010) traces the origin of the concept to forestry, meaning that one should not harvest more than the forest could yield in new growth. The call for a ‘sustainable global society’ was probably first expressed in the 1970s by the World Council of Churches, coupling justice, by correcting maldistribution, and ecology, pointing at humanities dependence upon the Earth (Langhelle, 2000). Du Pisani (2006) traces environmental sustainability to the idea of progress itself, and Dryzek (2013) links the concept to industrialisation, arguing that all environmental discourses embody a dissociation from industrial society in more or less radical ways. As a policy concept, it has its origin in the World Commission on Environment and Development (WCED) report ‘Our Common Future’ (Brundtland, 1987). In the Brundtland report, sustainable development is defined as development that: “meets the needs of the current generations without compromising the ability of future generations to meet their own needs” (Brundtland, 1987, p.15). Already a decade after the report, the term sustainable development had been deemed ‘dangerously vague’, ‘elusive’, ‘an oxymoron’ and ‘a cliché’.^6^ It has recently been criticised for being an ‘empty signifier’, which means that while appearing to address fundamental concerns, it means very little in particular and is subject to radically different interpretations (Brown, 2016). Nevertheless, the concept soon became the dominant expression of ecological concern (Dryzek, 2013). Sustainability is a concept that embraces social, economic and physical dimensions and the framing of the concept has centred more around three pillars, a development that Kuhlman and Farrington (2010) trace to Elkington’s Triple Bottom Line concept, indicating tightened relations between the ideas of sustainability and CSR. The three-pillar approach to is now embedded both in the Organisation for Economic Co-operation and Development (OECD) framework (Strange & Bayley, 2008) and by the UN. The Agenda 2030, where 17 sustainable development goals are launched, is promoted as a plan for action for people, planet and prosperity, adding peace and partnership (United Nations, 2015).

## The transformation of the CSR discourse in the public sphere

### The discursive approach

Discourses are always embedded in specific socio-political environments, and the main topics of CSR have altered in the course of time (Mark-Ungericht & Weiskopf, 2007). Plausible analysis of the discursive construction and transformation of CSR should therefore consider topics that has been vital for the debate on the business-society relationship in the period studied. The discussions on *sustainability* and *the social model* (the social organisation of work and welfare) are seen as particularly significant in this context. The aim of this study is to 1) examine the transformation of the CSR discourse in the public sphere and 2) interpret how and to what extent the concept of CSR has been influenced by the changing understanding of sustainability and the Norwegian social model during the last three decades. Thus, we are interested in understanding how CSR thinking has transformed in perspective of the discourse of the social model and the increased attention to sustainability. With this in mind, we focus not merely on interpreting the transformation of the CSR concept in itself, but also on the *intersection* between CSR and the concepts of sustainability and the Norwegian social model. Guiding our investigation is the general assumption that when discourses align over time, they can represent a formative power. It is, then, important to understand the force and direction of this formative power.

We see discourse as “an ensemble of ideas, concepts and categories through which meaning is given to social and physical phenomena, and which is produced and reproduced through an identifiable set of practices.” (Hajer, 1995, p. 44) This is a definition that emphasises that although meaning construction through discourse is a continuous affair and seemingly intangible, discourses are also material and possible to identify by tracing linguistic regularities or patterns of argument.

Three crucial assumptions guide our investigation. First, we presuppose that media has a role in the formation of public discourses and that media plays a role in the debates around CSR (Buhr & Grafström, 2007; Carroll, 2011; Schultz et al., 2013; Tang, 2012) Second, we see corporations and business communities as reality-shaping actors that contribute to social meaning, like any other communicating entity (Hajer & Laws, 2006; Schultz et al., 2013). By participating in public debate, representatives from the business community contribute to the emergence of certain world views, as do politicians and researchers. However, and this is the third point, such engagement must not be reduced to an expression of strategic behaviour or individual agency alone. Discourse analysis deals with larger meaning structures that emerges from the *interaction* of elements of discourse, as an ongoing play of contrasts and consonances (Wagenaar, 2011). Thus, shared meanings emerge partly by happenstance, partly on purpose and partly by convenience. Often, this goes on unnoticed by the people involved (Hajer & Versteeg, 2005). CSR is therefore making sense and giving sense to different actors in a dynamic and ongoing continuum of different and even competing meanings and narrations (Schultz et al., 2013).

### Method for searching the changing patterns in the public discourse

#### Data collection

The source for our data collection is the Norwegian newspaper Aftenposten. Aftenposten is the largest printed newspaper by circulation in 2018 and has been one of the top two dailies in Norway during the period of our study. It has positioned itself as rather conservative, leaning to the political centre-right (Nohrstedt et al., 2000). The newspaper is a non-tabloid, covering national and international news and regarded the most important arena for discussions on complex but popular issues covering all sectors. Other CSR scholars have used financial papers to construct their research database (Buhr & Grafström, 2007; Grafström & Windell, 2011; Herzig & Moon, 2013). However, our focus is on public opinion, not restricted to those with a devoted interest in the world of business. A broadsheet of high circulation is also more likely to represent general discussions on our two other themes, the social model and sustainability. As online papers had few readers in initial years, we focused on paper press.

**Fig. 1:**
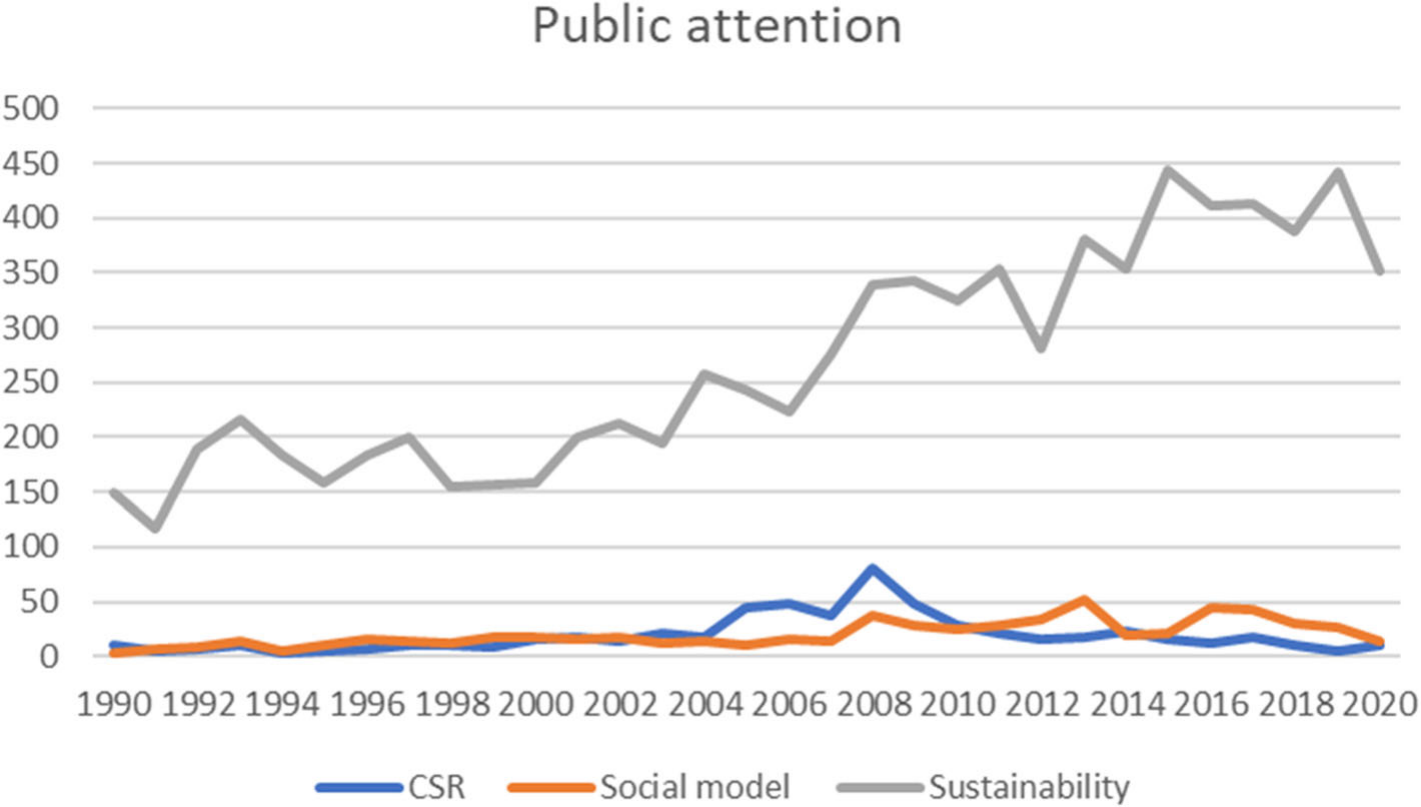
Graphical representation of search results. Database: Atekst. Data is collected using searches samfunnsansvar AND bedrift*, *“den norske modellen”*, and bærekraft* specified to the period 1.1.1990–31.12.2020 in the newspaper Aftenposten

To perform our search in Aftenposten we used the web-based database Atekst, which is the largest media database in the Nordic countries. To capture the transformation of the concepts during three formative decades, data was collected from 1.1.1990–31.12.2020. For an investigation of the three discourses, search has been conducted as suitable for each term. As an indication of the implicit nature of CSR in Norway and due to language issues, the English term CSR is rare in Norwegian vernacular, while social responsibility (“samfunnsansvar”) is common. To capture the understandings of social responsibility related to business, we used the Boolean operator AND for the words ‘social responsibility’ in combination with the word ‘business’ with truncation to include stemmed words, in Norwegian: samfunnsansvar AND bedrift*. This search brings 593 texts. Phrase search with quotation marks is performed for ‘the Norwegian social model’, *“*den norske modellen*”*, as the term employs this specific combination of words. The search brings 625 texts. The term ‘sustainability’ needs truncation in Norwegian. By searching for bærekraft* we capture the necessary discussions on sustainability, what is sustainable (or not), and also on the crucial concept of ‘sustainable development’. This search brings 8298 texts in Aftenposten. In total 9516 texts compose the material collected. We recognise that not all aspects and nuances of the individual discourses are captured by this selection. However, we believe it to be sufficient for our purpose. An overview of the frequency over time is presented in Fig. 1.

#### Data coding and analysis

Since our focus is on the formative process and transformation of the discourse, a quantitative assessment would not suffice. By reading and re-reading the texts, we were able to identify meaningful patterns and nuances and to eliminate data that was irrelevant to the study. Due to a large sample but nevertheless in accordance with the longitudinal approach, in-depth readings were concentrated to the years 1990 2000 2008 and 2018. The year 2008 was assumed to be of interest as this was the year of the financial crisis which posed a threat to the business system and possible changes in discourses (Herzig & Moon, 2013). The data were analysed using abductive research techniques based on thematic coding with adapted use of software support (Silver & Lewins, 2014). Theme-based coding involves identifying and examining patterns or themes within data that are important to the description of a phenomenon.

The in-dept reading and coding was split between the three authors. The first author read and coded the data concerning sustainability, the second author read and coded the data concerning the social model and the third author read and coded the data concerning CSR. Consistent with an abductive research technique, new themes were identified as they emerged. A criterion for determining a new theme can be described as the use of the term within a new social field or sector, e.g., the introduction and recurrent application of the word “sustainable” in connection with discussions on tourism or energy supply. No categories were produced in advance or common template made, as this would risk setting boundaries for coding and interpretation. Due to the large number of texts in the sustainability sample, coding for this material was performed using the software Nvivo. In addition, authors wrote short memos during in-depth reading and documented arguments and key concepts in summarised form, as well as insights and preliminary interpretations. After the completion of in-depth reading and coding summaries, categories and memos were revisited by each individual author to identify recurrent themes, repetitive traits or trends that provide understanding of discourse transformation. The analysis of the transformation within the three discourses will be presented separately in section Examining the discourse - Specified to CSR–Examining the discourse - Specified to Sustainability.

As the final step, the results of the three separate inquiries have been compared and discussed with specific emphasis on similarities and overlap and on differences and tensions. To remind the reader, our goal was not to study CSR discourse in isolation but also to unwrap the suppositional influence of the social model and sustainability discourses into the CSR discourse. These influences were discussed in terms of what they imply for the width, depth and level of CSR discourse, as introduced in Table 1 in the introduction, to better understand the comprehensiveness of the transformation that has taken place. This final analytical step confirms that discussions on CSR, sustainability and the social model often approach the same challenges and that the public discourse of CSR has transformed to become part of a broader societal discussion about sustainability. Our reflections on this will be further elaborated in section The Long-term Transformation of the Concept of CSR?.

## The long-term transformation of CSR discourse

### Search results

In statistical terms, sustainability is referred in the media substantially more often than the other two concepts, and this has been the case throughout the whole period studied. Through in-depth reading, it was established that a notable number of the early texts on sustainability did not fall within the subject matter of the study and were therefore irrelevant for our research question. As an example, sustainability is a word often used in the culture sections or obituaries in the beginning of the 1990s. Thus, a purely quantitative assessment does not suffice, however, the results suggest that there is reason to examine in what way the sustainability discourse in popular usage influences discussions on corporate responsibility and the relationship between business and society.

#### Examining the discourse - specified to CSR

We find that CSR and the social responsibility of companies got little attendance in the public debate in the 1990’s. This changes during the space of the 2000’s, but after a sharp increase around 2008, the attendance in recent years has again declined.

In the initial year studied, 1990, the understanding of CSR was eclectic and shows no coherent trend. The discussion varies from leadership, sports, maritime operation, rule of law and taxing regime, to mention some. In some articles there is a call for more responsible businesses in general, however, these articles do not define what is meant by ‘responsible’. In terms of depth, it looks as if we are at the bottom of Carroll’s pyramid. The tendency of low attention and little thematic consistency continues in 2000. When discussed, CSR is mainly referring to ethical behaviour. However, in 2000 we see the wake of an increasingly critical focus on business activities abroad. In particular, state-owned multinational companies are scrutinised for their operations in developing countries. Now, environmental sustainability issues are discussed, like activities in the rainforest, how sustainable food supply is, and more generally how companies behave in the global economy.

The strong presence of these discussions is noticeble in 2008, many even before the financial crisis, which materialised in the fall. At this point of time, the global telecommunication company Telenor was involved in a corruption scandal in India and Statoil Hydro’s operations in Brazil are debated. These events initiated a large discussion about leadership ethics and management judgement. A discussion about the teaching of ethics in business schools followed. The financial crisis caused a widening of this debate. As the severity of the event was recognised, several articles over the winter 2008 and 2009 call for a rethinking of global capitalism. However, interesting enough, to avoid recession, the Norwegian Minister of Finance asked people to increase consumption as part of showing individual responsibility.

The volume of articles displaying discussions on CSR increased considerably from the beginning of our study and peaked in 2008. After, the general public attention decreased towards 2018. CSR is again mentioned in relation to discussion of several and unrelated topics like culture, sport, construction industry, Trump presidency, media, shipping, Chinese intellectuals, and aviation. The concept is used mainly as a reference to being responsible in relation to ethics and to the larger society. Still, the wrongdoings of multinational companies abroad are a recurrent theme, set off by for example Hydro’s closing down of three production facilities in Brazil because of pollution. The debate reached a new level when a Professor of Economy at the University of Oslo, questioned the future of capitalism. In this discussion, the perspective has been system change towards a more socially sustainable future, rather than ethical responsibility by the individual firm. Also, the UN sustainability goals were now in focus. As for the new discussion, the #Me-too movement led to a shift where responsibility was taken in-house to all kinds of companies and organisations, not primarily the large, global ones.

Overall, the public understanding of CSR is not far from government policy application, where CSR has been interpreted as global and non-domestic, assimilated to Norwegian internationalist and humanitarian ambitions and traditions (Gjølberg, 2010). As a concept, it is fragmented and the attention shifts throughout the period studied, perhaps due to the implicit form and tacit norms that characterises CSR in some contexts (Looser, 2019; Matten & Moon, 2008). Even though the concept has no strong explicit position in Norwegian newspaper discussions, we argue that the understanding has become deeper and wider. Business is expected to do what is just and fair and to avoid harm, but also to actively display a broad social engagement for the common good, which implies a near maximum position of CSR according to Table 1, and CSR, as we will show below, is increasingly used interchangeably with sustainability.

#### Examining the discourse - specified to the social model

When considering CSR in the context of Norway, many of the elements associated with social responsibility, such as a focus on distribution of power, widespread employee participation, sustained employment, equality of income, and working conditions are subjects already attended to by the Norwegian model and institutionalised in national regulations. In order to encompass the public discourse regarding these themes, it is therefore relevant to also analyse the media discussions related to the ‘Norwegian social model’.

The subject has maintained a constant presence at a stable hushed level from 1990 until 2018 and peaks regularly in the year prior to general elections. The increased globalisation in the 1990’s as well as privatisation of public services, created a constant call for persevering the Norwegian social model. Thus, the subject matter approaches the themes of national economy, social development and welfare, and was often formulated as a commentary on political solutions to these subjects. The subjects of tripartite collaboration, the economic contribution of employees compared to the cost of publicly funded benefits and also business ethics in Norway compared to international business, are all themes related to social responsibility that appear to provoke references to the Norwegian social model.

In advance of the general election of 2009, the media discussion of 2008 was influenced by the political debate, where political ideology and the support of social benefits and welfare invoke recur as main themes. The number of articles featuring the subject of the Norwegian social model rises to 28 in this period and the discussions are dominated by welfare, economy and political policy, as well as ideology and ethics in work-life. An article that resulted in many follow-up articles and debate was titled ‘Den Norske Modellen’ (The Norwegian social model), written by a politician, sparking discussion regarding policy and ideology in a welfare state. This presence of the Norwegian social model in a political debate reiterates the suggestion that social responsibility is highly institutionalised in Norwegian society. The absence of a public conversation on corporate social responsibility regarding these issues reinforces the suggestion that CSR in a Norwegian context is somewhat unnecessary. Instead of voluntary business initiatives of CSR, the advanced Nordic welfare states presuppose social and environmental concerns to be the concerns of government (Brejning, 2016).

The most notable development in the discussions on the Norwegian social model is the increasing overlap in the subject of social responsibility and economic and social sustainability in the discussion regarding immigration and welfare. Environmental sustainability is not included in the debates, indicating that the social model is primarily expected to secure economic and social development without considering ecological limits or concerns. The fact that in the wake of the financial crisis the debate on the social model has not increased, will by us be interpreted as a partly due to the fact that the broader debate on sustainability has incorporated this social dimension.

#### Examining the discourse - specified to sustainability

The data on sustainability includes 150 articles and opinion pieces in 1990, 158 texts in 2000, 339 in 2008 and in 2018 the number has increased to 390. In quantitative numbers the use of the word ‘sustainable’ has increased throughout the whole period. There are noticeable changes in the way the term is used and a marked expansion in topics incorporated or related to the discourse.

In 1990 the dominant discourse was the discourse of sustainable development, combining concerns for economy and the environment. The discussion at large encompassed questions of development, first and foremost in developing countries, in a combination with concerns on a variety of environmental problems, like pollution, the ozone layer, biodiversity, energy consumption and climate change. The tone was distanced and impersonal, relying on experts and collective units. There were no references to CSR or the responsibility of individual firms in these debates, as sustainability was seen as the duty of governments, entire business sectors and international organisations. The positive atmosphere of global cooperation withered during the 90’s and in 2000 sustainability was mostly discussed in relation to national resource management of fish, forests, and animal populations. Government and its agencies, not firms, were still expected to be responsible for finding a proper balance between exploitation and conservation. However, implicit in the discussions were the business perspective, where natural resources were seen as a pool from which private actors can profit, which again will increase national wealth and competitiveness. Another form of resource management entered the stage in 2000; that of waste management and recycling. The field of economics had at this time adopted the term: *sustainable economy* was increasingly used as a synonym for a healthy economy, which in essence meant a growing economy. Sustainable economy has since often been used in relation to the stock market or the survival of business sectors or firms. In 2008 there was a noticeable turn towards climate change and related issues. This meant that energy, transport, and carbon emissions became central. Biofuel, environmental technology, cities, ‘sustainable architecture’ and ‘sustainable tourism’ were related, new themes. A larger debate on the future of the Norwegian oil and gas industry spread. At this point, focus also moved to some extent from the authorities and onto the producer and the consumer, where Fairtrade or other certificates and labels, ecological food and sustainable consumption were interpreted as examples of a trend of individualising and nationalising the sustainability discourse.

Another new theme involved the rapidly expanding industry of salmon farming. When Marine Harvest fired 1.000 workers in Chile because of diseases in salmon farms, CSR and environmental aspects clashed: environmental damage caused bad economic results for the company, which again lead to social costs for the Chilean workers. Seeing the economy and the environment as two sides of the same coin came to characterise the discussions on climate mitigation and an Ecological Modernisation discourse (Dryzek, 2013; M. Hajer, 1995) gradually succeeded the sustainable development discourse in the material studied. This discourse promotes the ‘green economy’ and technological optimism where environmental problems become the burden of bureaucrats, technicians, and business. This shift relates to the CSR concept, as it gradually established the idea that the private sector will solve environmental problems by inventing new things and profit at the same time.

It must be remarked that critique against ‘non-sustainable’ behaviour has been stronger and more frequent since 2008. In the data we observed increased accusations of greenwashing; that is, the allegation that companies´ claims on environmental or social issues diverges from actual practice (Gatti et al., 2019).

To be noted, starting in 2008 and continuing with strength in 2018 there was a marked turn towards using the term sustainability in relation to social issues. ‘Sustainable welfare’ was made a central part of the centre-right government political platform. Here, economic growth, inclusive working life, poverty reduction, welfare arrangements, integration of minorities *and* climate crisis are main points, showing a melting pot of themes that makes the foundation for a sustainable welfare state. At the end of the study period there are recurrent references to *social sustainability*. These are themes that connects with parallel discussions on the Norwegian social model.

In 2018 plastic pollution and marine life has an upturn in attention alongside the almost all-encompassing focus on climate change and low carbon futures. Norwegian companies in oil and gas, fisheries, salmon farming and sea transport are portrayed as vital contributors to saving the sea by doing business responsibly helped by knowledge and new technical solutions. The EAT initiative couple food and climate and follows logically the idea of ethical consumption and change in eating habits, primarily reducing meat consumption. The international perspective which characterised the 90’s is reduced to single case stories and obedient and superficial references to the UN goals. A more critical counter-discourse gain strength towards the end of the period, mostly addressing environmental concerns and disbelief in current policies, both as case-to-case engagements and as critique of the socio-economic structures that encourages un-sustainable actions.

Overall, the sustainability discourse is amplified but progressively fragmented throughout the last three decades. Climate change, urban solutions, consumer and producer responsibility, technological ‘green’ innovation, robust financial markets, and a marked concern for the social welfare system can form a very brief summary of the contemporary stage in the evolution of the concept. This forms a contrast to the discourse of sustainable development in the 90’s and suggests a move in public attention from intergovernmental cooperation, foreign aid and fair distribution toward company and consumer.

### The long-term transformation of the concept of CSR?

In this article we use Norway as a case to elucidate the long-term transformation of the concept of CSR in public debate in a context where implicit and intrinsic CSR is assumed to characterise the business-society relationship. Our concern has not been the CSR concept itself, but rather the phenomenon of corporate social responsibility and the understanding of the business-society relationship in the public sphere. Norway is a market economy marked by strong social institutions and a collaborative structure between Government, business, and social partners. Therefore, CSR has been seen as an inherent aspect of Norwegian business-life. Referring to Table 1, CSR studies suggest that Norway has never embraced the marked economic thinking inherent in the Friedman doctrine. It has always been emphasised that business responsibility goes beyond maximising profit. The default position has been to engage with the social and political regime, and at the same time consider social and environmental aspect beyond profit. This means that the investigation of CSR discourse cannot logically be isolated from discourses on social and environmental aspects. However, from our analysis of the public discourse we observe that in the early phase; that is, in the 1990s, the CSR concept was to a large extent referred as an international and American debate. In the 2000, the concept more and more became flavoured by the Norwegian context. We argue that potential for change is heightened when separate discourses come together over time, under the premise that they share some common ideas. In our analysis of the discourses concerning CSR, the social model and sustainability, we see parallels and entanglements between discourses. The inference being that the long-term transformation of CSR discourse is influenced, making it increasingly comprehensive in level, width, and depth the last three decades. This reinforces a position in the right column in Table 1 where business is expected to engage in improving the social and political regime, in addition the increased focus on sustainability has encouraged a shift towards becoming agents of sustainability transformation.

It is this movement that forms the foundation for the argument that CSR has been subject to a long-term transformation in public discourse. When the term was used in the early 1990’s and early 2000’s, it was mainly to emphasise unethical behaviour and non-compliance with established norms. Business was supposed to comply with social norms and rules. When it came to the few examples of international corporations breaking norms, such as social dumping, corruption, or other aspects of globalisation, these were not generally treated as internal corporate problems, but problems that should be addressed through additional government regulation.

The discourse changes towards 2018, in this respect. Now it is the social and political regime itself that is in focus and the future of the socio-economic system and global capitalism is debated in addition to the social responsibility of individual firms. In the public sphere, the concept of CSR is somewhat absent in the subjects related to the social model. On face value, this could be taken to mean a public disinterest in the connection between business and social structure. Rather, the opposite is the case; with regard to typically CSR related subjects as employment, working conditions and welfare, the social model is taken for granted as a stable structure that deals with social concerns in harmony with business. The findings are consistent with the idea of business *in* society, juxtaposed to business *and* society (Freeman, 2013; Siltaoja & Onkila, 2013), and literature on CSR in Scandinavia. Some may argue that this conclusion seems to contradict other recent testimonies of CSR becoming more explicit in Norway and Scandinavia (e.g., Carson et al., 2015; Tengblad & Ohlsson, 2010). In our opinion it does not, since these studies concentrate on business reporting and company self-representation, not public discourse. Our study simply proposes that the proposition of a more explicit CSR practice and self-representation in Norwegian companies seems not to have affected the public discourse in our material to a noticeable degree. The exception is in cases of greenwash accusations, where the explicit accounts of firms are met by critical voices. Such tensions are pointed out to likely occur when the implicit Nordic approach is confronted with the demands to more explicit approaches (Morsing & Strand, 2014). Furthermore, we think that the discourse on the social model seems to follow some of the same pattern as CSR, namely, to be integrated into the larger discourse on sustainability. This, we argue, indicates that the pattern we see is more general. Subsequently we will expect that the Norwegian case points to a more general trend where responsibility of business must be seen in a larger perspective of society’s sustainability challenges.

Our data gives support for arguing that the increased attention to sustainability has challenged business behaviour. In line with development in the sustainability discourse, businesses are increasingly envisioned as drivers of the green transformation. Considering environmental sustainability, businesses are seen as both a part of the problem and as part of the solution. This recognition has gained momentum after the Paris agreement. Critique of unsustainable business conduct constitute the most tangible aspect of problematisation within the sustainability discourse and is articulated alongside an increasingly comprehensive and complex range of sustainability concerns. From a long-term perspective, sustainability discourse has emerged from a marginal position in the public debate to become a major factor in setting the agenda for business policy and strategy. The sustainability discourse can thus be interpreted to make explicit a wide range of responsibilities and concerns on behalf of business actors which the CSR discourse does not articulate with equal clarity. In this regard, the sustainability discourse both influences the transformation of the CSR discourse and to some extent outrivals it.

## Conclusion

In this paper we have investigated the long-term transformation of the public CSR discourse in Norway as it has been articulated in media texts from 1990 to 2020. Through thematic categorisation and in-depth reading of texts that discuss CSR, the social model and sustainability, we have shown that discourse on the subject matters have evolved from relatively dispersed discussions in the 1990s to increasingly entangled over the three decades, aligning CSR with an increasingly comprehensive concept of sustainability. In the Norwegian media, the sustainability discourse has progressively permeated discussions on the business-society relationship. Although there is an increased attention to CSR in the academic literature (Carroll, 2021; Fernández-Gago et al., 2020), it is interesting to observe a decreased reference to the term in public discourse over the last decade. However, even if discussions on CSR and the social responsibility of business have declined since 2008 in our data set from the newspaper Aftenposten, we argue that the concept of CSR has become more ambitious as a result of inherent premises in the sustainability discourse, meaning that the public understanding of the role of business in society envisions a wide and deep engagement where business actors are expected to be proactive agents of social and environmental change. The study empirically confirms the intuitive presupposition that how we think about CSR is increasingly affected by the sustainability discourse. The implication of this is that the discussions on corporate social responsibility should not construct a divide between business and society as the Friedman doctrine proposes but allow a broader debate. Our study adds to the discussion of the long-term transformation of the concept of CSR. The main conclusion we draw from our study is that in public discourse, the increased attention to sustainability has become an overriding concern that has changed the way CSR is discussed. At least in an integrated CSR, it has reduced the split between business and society, paving the way for new, integrated dialogue. How this will affect the practices of business in relation to CSR is yet to be seen.

The limitations of our study are found in that we attempted to gain overview, which means that some details and perspectives are omitted. For instance, we have not identified the main actors that has contributed to discursive changes, neither have we studied whether changes in public discourse have had impact on motivation, behaviour, self-representation, or other aspects of corporate culture. This could be suggestions for further research. In addition, since discourses are open, there are other important contextual changes which may have influenced the transformation of CSR discourse but which we did not include in our analysis. The Covid 19 pandemic represents an enormous contextual alteration that may have important influence that could be subject for a future study. In addition, the qualitative method applied involves interpretation which means rendering the data meaningful in certain ways and not others (Gee, 2010). In order to secure validity to the interpretations, findings have been compared and discussed repeatedly by the three authors.

A similar study can be performed in other countries to expand knowledge on public CSR discourses in relation to sustainability and the socio-political system. It would be interesting to see if there are similar changes of patterns in the public discourse in other countries. Also, it would be interesting to know of the general trend in such changes deviate between contexts of varieties of capitalism. Thus, a key question for further research that our study might have provoked, is whether the increased attention to sustainability have widened the gap between intrinsic CRS thinking in coordinated economies, in comparison with more extrinsic CSR in liberal marked economies.

## Data Availability

Texts analysed is retrieved from the electronic database Atekst, which is a newspaper archive for Norwegian and Swedish newspapers. It is a paid service, and subscription is handled by the University of Agder. Link to atekst: https://web.retriever-info.com/services/archive

## Abbreviations

CSR: Corporate social responsibility
EU: European Union
GDP: Gross domestic product
IMF: International Monetary Fund
OECD: Organisation for Economic Co-operation and Development
UN: United Nations
US: United States
WCED: World Commission on Environment and Development

## Footnotes

1 This general picture is supported by the study of Madsen and Stenheim (2014).

2 The Netherlands being included in the Nordic regional cluster due to the observed similarities in adopting co-responsibility and partnerships (Maon et al., 2017).

3 This was acknowledged by Matten and Moon in the 2008 contribution.

4 The formal name is Norwegian Government Pension Fund. For continuously updated fund rankings see: http://www.swfinstitute.org/fund-rankings/.

5 The use of the geographical term “Nordic” normally refers to the five countries of Norway, Sweden, Finland, Iceland, and Denmark. The term “Scandinavia” normally refers to the three countries of Norway, Sweden and Denmark. For an elaborated description see Strand et al. (2015)

6 For a summary of early critique, see Mebratu (1998).
